# A Passive Testing Approach for Protocols in Wireless Sensor Networks

**DOI:** 10.3390/s151129250

**Published:** 2015-11-19

**Authors:** Xiaoping Che, Stephane Maag, Hwee-Xian Tan, Hwee-Pink Tan, Zhangbing Zhou

**Affiliations:** 1 School of Software Engineering, Beijing Jiaotong University, Beijing 10044, China; 2 Telecom SudParis, SAMOVAR, CNRS, Universite Paris-Saclay, Evry 91000, France; E-Mails: stephane.maag@telecom-sudparis.eu (S.M.); zhangbing.zhou@gmail.com (Z.Z.); 3 School of Information Systems, Singapore Management University, Singapore; E-Mails: hxtan@smu.edu.sg (H.-X.T.); hptan@smu.edu.sg (H.-P.T.); 4 School of Computer and Communication Engineering, University of Science and Technology Beijing, Beijing 100083, China

**Keywords:** passive testing, wireless sensor networks (WSN), conformance testing

## Abstract

Smart systems are today increasingly developed with the number of wireless sensor devices drastically increasing. They are implemented within several contexts throughout our environment. Thus, sensed data transported in ubiquitous systems are important, and the way to carry them must be efficient and reliable. For that purpose, several routing protocols have been proposed for wireless sensor networks (WSN). However, one stage that is often neglected before their deployment is the conformance testing process, a crucial and challenging step. Compared to active testing techniques commonly used in wired networks, passive approaches are more suitable to the WSN environment. While some works propose to specify the protocol with state models or to analyze them with simulators and emulators, we here propose a logic-based approach for formally specifying some functional requirements of a novel WSN routing protocol. We provide an algorithm to evaluate these properties on collected protocol execution traces. Further, we demonstrate the efficiency and suitability of our approach by its application into common WSN functional properties, as well as specific ones designed from our own routing protocol. We provide relevant testing verdicts through a real indoor testbed and the implementation of our protocol. Furthermore, the flexibility, genericity and practicability of our approach have been proven by the experimental results.

## 1. Introduction

The phenomenon whereby data are acquired from several network-connected sensors for the purposes of sense-making and intelligent decision-making in smart systems is drastically accelerating. It is expected that there will be increasing numbers of large-scale deployments of devices that are instrumented with multi-modal sensors in the near future (e.g., in cities, transportation, museums, energy systems, *etc*.). These sensor devices generally have small form factors and possess limited processing and storage capabilities. In addition, they are typically powered by limited battery supplies and/or energy-harvesting sources.

An essential component for the realization of these wireless sensing devices is the data transport service, *i.e*., the ‘routing protocol’, to carry data from the sensor devices to the back-end servers via one or more (Internet-enabled) gateways, for further processing and analysis. Due to the energy constraints of the nodes and the limited transmission range, the IEEE 802.15.4 standard [1] for low-rate wireless personal area networks (LR-WPANs) is often used for inter-nodal communications. As the transmission range of such a radio is typically limited (often in the range of tens of meters), nodes that are deployed over large spatial regions are expected to communicate to the gateway(s) via multiple hops.

Due to these aspects and the few resources of the sensor nodes, providing efficient and reliable routing protocols is an important and challenging problem. For years now, several routing protocols for wireless sensor networks (WSN) have been standardized and developed [2,3,4,5,6,7,8,9,10,11]. While many efforts have been produced for their development, integration and performance evaluation, the conformance testing of their implementations has been somehow neglected.

The conformance testing of routing protocols is crucial, as a data transport service that does not behave in its expected manner can have detrimental impacts on data collection and severely impact the overall quality, as well as the usefulness of the system. Furthermore, in large-scale sensor systems, a single bug fix can be an extremely time-consuming and labor intensive process, especially if the nodes are placed in inaccessible locations across varying spatial locations.

However, though the conformance testing of these protocols is crucial to the reliability of the systems, there are paradoxically very few works. Indeed, the major ones are mostly dedicated to their performance analysis, application and security studied through simulation or emulation relying on non-formal models [12,13,14,15,16,17]. We propose in this paper a formal syntax and semantics to express the requirements of WSN routing protocols and to passively test them on real execution traces. These syntax and semantics do not need to be inevitably used on top of any protocol standard. We herein model and test common WSN requirements, as well as specific ones designed from a novel non-standardized routing protocol. We show that our approach is helpful from the very first stage of the system development life cycle and is complementary to the normalization phase, as well.

There are two main ways for testing the conformance of a protocol, either in an active or passive way. While the active ones require a simulation of the implementation under test (IUT) and an important testing architecture, the passive ones are based on the observation of input and output events of an IUT at run-time. Basically, passive testing techniques are applied whenever the state of an IUT cannot be controlled by means of test sequences, either because access to the interfaces of the system is unavailable or a reset of the IUT is undesired. This is specifically the case with the WSN where the topology is dynamic and the testing interfaces not always available. Moreover, in actively testing WSN protocols, we often deal with no direct access, wide fluctuations in the wireless channel and a high dependence on prevailing environmental characteristics.

In this context, some passive techniques have been proposed to test the conformance of protocols in wired systems. These passive testing approaches are based on the record of the observation during run-time and its comparison with the expected protocol behavior defined by either a formal model or as a set of formally-specified properties obtained from the requirements of the protocol [18]. The observation is performed through a set of points of observation (PO) on monitored entities composing the system under test. The protocol messages observed in execution traces are generally modeled and analyzed through their control and data parts [19]. These approaches are efficient, but are not suitable for wireless environments, like WSN and its own inherent constraints, as mentioned above. Although we already studied and tested wired networks with our formal testing approach [20], we present in this paper an adaptation of this approach to passively test a routing protocol in wireless sensor-based smart systems. Common and specific WSN protocol properties are evaluated on collected execution traces to provide testing verdicts. We successfully assess our approach on an indoor testbed. As far as we know, this is the first work on formal passive testing of routing protocols in a WSN.

Our paper’s primary contributions are:

- A logic-based approach for formally specifying some functional requirements of the WSN routing protocol. We provide an algorithm to evaluate these properties on protocol execution traces that are collected at run-time through PO set on the nodes.
- With a real indoor testbed and the implementation of a new routing protocol and the implemented algorithm, we demonstrate the efficiency and suitability of our approach by providing relevant testing verdicts. The modeled requirements do not need to be compulsorily standardized, which helps the development life cycle processes.

The rest of this paper is organized as follows: Section 2 discusses existing work in the literature. We describe the formal approach of our passive testing framework in Section 3 and provide details of the sensor networking protocol to be tested in Section 4. Section 5 contains details of the experimental setup and evaluation results. We conclude our work in Section 6.

## 2. Related Works

Although there exists many studies on protocol evaluation in WSNs, they mostly tackle their performance, application or security issues [12,13]. Very few works focus on the conformance testing of these protocols. In the following, we present the ones that inspired us and compare them.

In [21], the authors study common functional testing approaches to tackle the security issues in wireless sensor networks. Since conformance testing techniques cannot assess the efficiency of the implementation of a security protocol (mainly because most of the security concepts are close to non-functional mechanisms), the authors propose to add randomness tests to conformance tests dedicated to the evaluation of security functions. One part of the SPINS protocols (security protocols for sensor networks) is chosen to illustrate their approach.

Like in the previous proposal, [22] recently proposed a remote testing method by stating the design and development of test routers in WSN. For that purpose, a remote testing platform is developed and assessed. The authors tackle the bad environment in which nodes are intensively placed. They study the energy consumption and the dynamic change of the network topology. These inherent constraints often prevent the testers from meeting the tested protocol requirements. Still, with the same objectives, a formal testing approach is proposed in [23]. A nodes’ self-similarity approach for testing wireless mobile *ad hoc* networks (MANET) is presented. Formal specifications and emulations are applied through the Dynamic Source Routing (DSR) [24] protocol to provide relevant verdicts about the test of one of its implementations.

We also got inspiration by [25] in which real wireless sensor nodes are deployed to test, through virtualized emulated nodes, TCP and some functions of their own faulty routing protocol RMRP (Rime mesh routing protocol). A state model is defined and test suites generated and executed through their architecture based on an interesting generated dynamic topology. In recent papers [26,27], the authors provided a suitable open conformance test system for standard-based WSNs, dedicated to the protocol evolution and the various hardware interfaces of the sensor nodes. However, these approaches do not provide a way of modeling non-standardized requirements as we tackle in this work. In our work, we propose an approach to test the functional behaviors without using any formal specifications. Further, we do not claim to interact with the designers through any testing process, but just by receiving the expected protocol requirements.

Though interesting results have been obtained by the works mentioned above, the tests are mostly applied through active testing architectures. The tests are intrusive and need to generate important test suites. One of our challenges is to the contrary to avoid any interaction with the implementation. By passively testing the implementation protocols, we also do not need any testing scripts and the related specification stages. Moreover, we here work on a real testbed, avoiding the simulation or emulation drawbacks often met in these kind of studies.

Recently, interesting works on passive diagnostics in WSN have been presented. First, we may cite [28] in which the authors propose a probabilistic diagnosis approach for inferring the root causes model of abnormal phenomena in wireless sensor networks through the passive observation of eventual symptoms. A light-weight packet-marking scheme is defined to collect relevant hints and the probabilistic inference model located at the sinks targets the expected hints. Second, [29] proposes a passive observation and mining of relevant collected information to diagnose WSN performance failures. The authors’ technique deduces the root causes of failures by focusing on the relationships between the sensing data, the failures in the networks and a failure knowledge library. Furthermore, [30] proposes passive distributed assertions (PDA) as a novel tool for identifying failure causes. It allows a programmer to assert certain predicates over a distributed node state, while minimizing interference with the sensor network. Finally, a combination of active and passive testing approaches is presented in [31] for fault localization in WSN. In this work, the scope is different from ours. The faulty components are searched by optimal end-to-end test suites, whereas we test the conformance of the protocols without trying to point at the faulty entity, since the implementation of the tested protocol is our objective. However, the proposed passive observation and the data aggregation procedure are of high interest in the definition of our formal approach.

We got inspired by these above-mentioned approaches, even if they are applied to the diagnosis or fault localization of WSN. Indeed, their symptoms or failure signatures can be compared to our formal properties, although in our framework, a logic-based approach is used. Moreover, some correlation processes are commonly applied in passive testing techniques. Nevertheless, we do not need any inferred models and do not impact the nodes reliability by any active processes. Additionally, different from passive distributed assertions, which concentrate on verifying the program states, we focus on a different issue: testing the behavior of the system, whether it conforms to the specific requirements.

## 3. Formal Passive Testing Approach

### 3.1. Preliminaries

We here define the syntax allowing us to describe some functional properties of network protocols.

**Definition 1.** *A message of a protocol P is any element $m\in M_{p}$.*

For each $m\in M_{p}$, we add a real number $t_{m}\in R^{+}$, which represents the time when the message *m* is received or sent by the monitored entity. The data domains are defined as atomic or compound. Once given a network protocol *P*, a compound domain $M_{p}$ can be defined by the set of labels and data domains derived from the message format defined in the protocol specification/requirements.

**Definition 2.** *A term is defined in Backus–Naur form (BNF) as term::=c∣x∣x.l.l...l, where c is a constant in some domain, x is a variable, l represents a label and x.l.l...l is called a selector variable.*

**Definition 3.** *An atom is defined as the relations between terms, A::=p(term,...,term)∣term=term∣term≠term∣term<term.*

**Definition 4.** *A clause is an expression of the form $A_{0}\leftarrow A_{1}\wedge ...\wedge A_{n}$, where $A_{1},...,A_{n}$ are atoms. The relations between atoms are stated by the definition of clauses.*

**Definition 5.** *A formula is defined by the BNF: $\phi ::=A_{1}\wedge ...\wedge A_{n}$∣ϕ→ϕ$|\forall_{x}\phi$$|\forall_{y>x}\phi$$|\forall_{y<x}\phi$$|\exists_{x}\phi$$|\neg \exists_{x}\phi$$|\exists_{y>x}\phi$$|\exists_{y<x}\phi$, where ∃ and ∀ represent “it exists” and “for all”, respectively.*

According to these definition, we define the syntax based on the basic unit term. The ¬ quantifier is newly introduced to formalize some specific condition in the WSN. In the following subsection, we introduce the semantics and algorithm used for our testing approach.

### 3.2. Semantics and Algorithm

The semantics used in our work is related to the traditional Apt–Van Emden–Kowalsky semantics for logic programs [32], from which an extended version has been provided in order to deal with messages and trace temporal quantifiers.

**Definition 6.** *A substitution θ is a finite set of bindings $\theta =\{x_{1}/term_{1},...,x_{k}/term_{k}\}$, where each $term_{i}$ is a term and $x_{i}$ is a variable, such that $x_{i}\neq term_{i}$ and $x_{i}\neq x_{j}$ if i≠j.*

Given a formula *ϕ* defined by using a set of clauses *K* as we mentioned above, a satisfaction result ‘⊤’ (‘pass’), ‘⊥’ (‘fail’) or ‘?’ (‘inconclusive’) will be given for a particular trace *ρ*. Using substitution *θ*, we recursively evaluate the formula *ϕ* (*i.e*., protocol property) on the real protocol execution trace *ρ*, coupled with a modification of SLD (selective linear definite-clause) resolution algorithm [33] for the evaluation of Horn clauses.

The evaluation process is described as follows:

$$eval\left(A_{1}\wedge ...\wedge A_{k},\theta ,\rho \right)=\left\{\begin{array}{cc} ⊤ & \begin{array}{c} \text{if}\;A_{1}\wedge ...\wedge A_{k}\theta \\ \text{has}\;\text{a}\;\text{result} \end{array}\\ ⊥ & \;\text{otherwise} \end{array}\right.$$

For every possible value *x* in the trace, the following formula is used:

$$eval\left(\forall_{x}\phi ,\theta ,\rho \right)=\left\{eval\left(\phi ,\theta \cup x/m,\rho \right)|\forall_{m}\in \rho \right\}$$

If the formula $\forall_{x}\phi$ is included in another formula, the result of evaluation is provided by:

$$eval\left(\forall_{x}\phi ,\theta ,\rho \right)=\left\{\begin{array}{cc} ⊥ & \begin{array}{c} \text{if}\;\exists_{m}\in \rho \;\text{where}\\ eval(\phi ,\alpha ,\rho )=⊥\;\text{with}\\ \alpha =\theta \cup x/m \end{array}\\ \text{?} & \;\text{otherwise} \end{array}\right.$$

The result “⊥” represents any violation that has been found. The evaluation of $\exists_{x}$ is quite similar to the $\forall_{x}$, but it is looking for a “⊤” result.

$$eval\left(\exists_{x}\phi ,\theta ,\rho \right)=\left\{\begin{array}{cc} ⊤ & \begin{array}{c} \text{if}\;\exists_{m}\in \rho \;\text{where}\\ eval(\phi ,\alpha ,\rho )=⊤\;\text{with}\\ \alpha =\theta \cup x/m \end{array}\\ \text{?} & \;\text{otherwise} \end{array}\right.$$

The evaluation processes for $\forall_{y>x}$, $\exists_{y>x}$ and ϕ→φ are the same as described before; we will not repeat them here. The interested readers can have a look at our previous work [20,34].

We also provide the evaluation algorithm in the following.


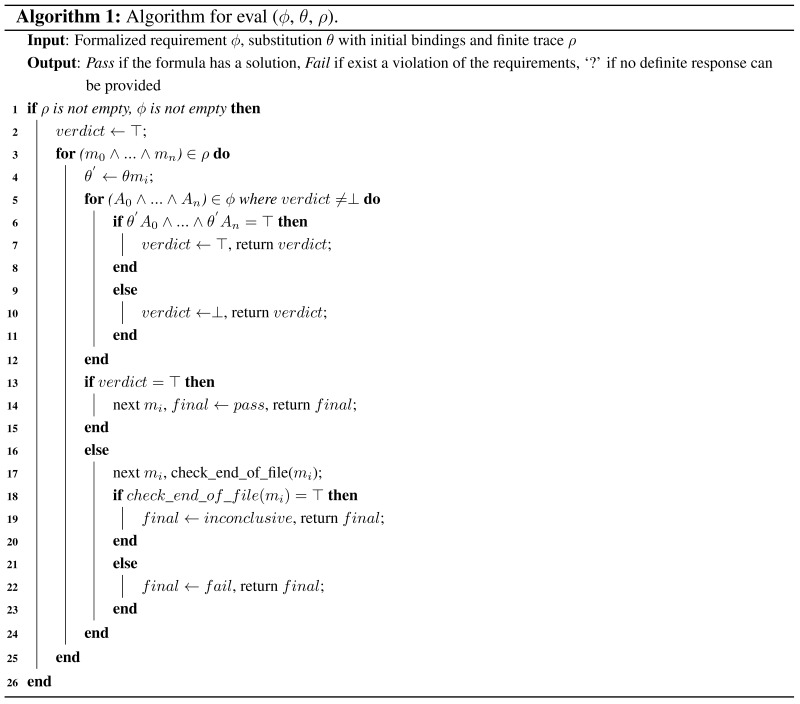


The algorithm starts by checking the existence of a trace *ρ* and a requirement *ϕ*. Then, if *ϕ* contains subformulas, they will be sequentially tested by using recursive calls. For testing a formula *ϕ* on a finite trace *ρ*, the algorithm will firstly assign the values to substitution *θ* from each message *m* in the trace. Then, the obtained $\theta^{{}^{\prime }}$ will be used to compare with each atom in the formula *ϕ*. If all of the atoms in *ϕ* are satisfied, a truth value ‘⊤’ will be assigned, and the algorithm will stop to test the next message *m*. Otherwise, any violation of the atoms will result in a truth value ‘⊥’, and the algorithm will immediately terminate the comparing process and stop to test the next message *m*. The truth values ‘⊤’ and ‘⊥’ will be eventually transformed to the verdict ‘pass’, ‘fail’ or ‘inconclusive’ as the semantics defined in Section 3. Finally, a final report will be provided when all of the sub-formulas of *ϕ* are tested through the trace *ρ*.

The complexity of the algorithm is decided by the number of quantifiers used in the formula being tested. In the worst case, the time complexity of our algorithm with *k* quantifiers is $O(n^{k})$ to analyze the trace, where *n* represents the number of messages in the trace. In the following section, we present a newly-designed routing protocol to be tested. It has been specified, developed and integrated in a real indoor testbed.

## 4. Protocol Description

We have designed our proprietary end-to-end networking stack for large-scale WSN deployments with modular components that are configurable, extensible, as well as plug-and-playable, depending on the sensing application requirements and the physical environment of the sensing region of interest. It is designed for scalability through the use of lightweight protocols that minimize both communication and storage overheads. Existing networking protocols are often designed in silo and, thus, unable to support our multi-faceted sensor network testbed requirements. In the following, we briefly describe its basics, the route establishment, the data transmission and the packet format.

The indoor testbed we use in this paper serves as an evaluation platform for our actual sensor-based smart system deployed in outdoor urban areas. As said before, our passive testing technique can be applied all along the development lifecycle, helping thus the normalization, as well as the deployment phase. Our outdoor sensor networks necessitate the use of multiple gateways in the network, so that timely and reliable data delivery can be achieved. Commercially available network protocols, such as ZigBee, are based on the *Ad hoc* On-Demand Distance Vector (AODV) routing protocol [35] and do not have inherent support for the use of multiple gateways in the network. The routing protocol in use is multi-hop and opportunistic in nature. It associates a metric called a gradient to each sensor towards the gateway of the network. According to prevailing link quality, a dynamic update of the routing gradients is performed.

### 4.1. Basics

A BEACON is periodically broadcast by each node (sensor and gateway) throughout its network lifetime and is used for:

1. Neighbor discovery
2. The estimation of link quality between neighboring nodes
3. The update of gradients throughout the network

It contains the:

1. Source node identifier
2. The monotonically-increasing sequence number for the detection of expired links and the prevention of routing loops
3. The gradient towards the gateway

During network initialization, each sensor node has a gradient value of infinity. The gateway then updates its gradient to be zero and sends this information together with its current sequence number, in its next BEACON. Upon receiving a non-infinite value of the gradient in a BEACON from a neighboring node $v_{j}$, the sensor node $v_{i}$ updates its gradient (according to the selected metric) if the following conditions are satisfied: (i) the estimated link quality between $v_{i}$ and $v_{j}$ is above a pre-defined threshold T; and (ii) the gradient of $v_{j}$ is smaller than the gradient of $v_{i}$.

When a sensor node $v_{i}$ has a DATA packet to forward to the gateway, the packet is broadcast into the wireless medium together with the gradient of $v_{i}$. A neighboring node $v_{j}$ that receives the DATA packet from $v_{i}$ will send an acknowledgment(ACK) and help to forward the packet only if the gradient of $v_{j}$ is smaller than that of $v_{i}$. The process is repeated along each hop until the gateway receives the DATA packet. It is thus possible for the gateway node to receive multiple copies of the DATA packet.

### 4.2. Route Establishment

At periodic intervals of 30 s, each node broadcasts a BEACON message to its one-hop neighbors for the purpose of neighbor discovery, topology maintenance, time synchronization and route discovery to the gateway. The BEACONs are used to compute running estimates of the link qualities between each node and its one-hop neighbors. This estimated link quality is subsequently used to determine the forwarding set from the node to the gateway.

Each sensor node is associated with a gradient (metric) towards the gateway node. The metric of an arbitrary node $v_{i}$ is denoted as $m_{i}$. Generally, packets flow from nodes with higher gradients to nodes with lower gradients. In our testbed, the gateway $v_{g}$ is initialized with a metric value of $m_{g}=0$; all other nodes in the network are initialized with a default invalid metric value of −1. This metric information is piggybacked in the BEACONs that are transmitted by each node.

Upon receiving a BEACON with a valid metric value (*i.e*., $m_{i}\geq$ 0) from an arbitrary neighbor $v_{i}$, each node $v_{j}$ will update its metric value $m_{j}$ to the gateway as follows: the metric of node $v_{j}$ through the use of neighboring node $v_{i}$ is given by $m_{j}\left(i\right)=m_{i}+\Delta$, where $\Delta \in {ℜ}^{+}$. If the estimated link quality between $v_{i}$ and $v_{j}$ is above a pre-determined threshold *T* and the metric through $v_{i}$ provides a better gradient to the gateway than the current metric (*i.e*., $m_{j}\left(i\right)<m_{j}$), $v_{j}$ updates its metric, such that $m_{j}=m_{j}\left(i\right)$. In our indoor testbed, we use the values of Δ=10 and T=80%. Due to the transient nature of the wireless links in the network, the neighbor list and metric of a node can expire if the node does not receive BEACONs from its neighbors after a period of time.

### 4.3. Opportunistic Data Transmission

Data transmission takes place in an opportunistic fashion within the network. A node $v_{j}$ with data to send to the gateway will include its current metric $m_{j}$ in the DATA packet, which is broadcast to the local one-hop neighborhood. An arbitrary neighbor $v_{i}$ that receives the data packet will send an acknowledgment packet ACK to $v_{j}$, if it has a better metric to the gateway, *i.e*., $m_{i}<m_{j}$. As wireless links may fluctuate, the transmitting node $v_{j}$ can retransmit DATA packets up to a maximum of five times before the packet is discarded from its transmit buffer. The process is repeated along each hop until the gateway receives the DATA packet. Due to the opportunistic nature of the routing protocol, it is possible for duplicate copies of the same packet to arrive at the gateway by using sequence numbers. The duplication of packets is resolved at the back-end through the use of sequence numbers.

### 4.4. Protocol Format

There are three possible types of packets that are used in the protocol:

- BEACON packets that are broadcast to neighboring nodes at periodic intervals of 30 s.
- DATA packets that are generated by the sensor nodes at periodic intervals of 120 s.
- ACK packets that are generated by the gateway or intermediate forwarding node (*i.e.*, the node that receives the data from another node with a higher metric).

The corresponding format of each packet is as follows:

- BEACON: [sequenceNum], [UNIXTime], [gatewayAddress], [gatewaySequenceNum], [metricToGateway]
- DATA: [portNumber], [dataLength], [metricToGateway], [dataSequenceNumber], [routeIncluded]
- ACK: [dataSource], [dataSequenceNumber]

**Example 1.** *Transmitted message (BEACON): 105, 65535, 0, 3, 9, 1404388288, 101, 1404388247, 30.*

In Example 1, we illustrate a BEACON packet of sequence number nine, that is broadcast by node 105 to its neighboring nodes at time 1404388288. The BEACON contains the information that the metric from node 105 to the gateway 101 is 30 and that the last known gateway sequence number from 101 is 1404388247. The gateway sequence number is used to expire stale routes and prevent routing loops in the network. We present in the following the testing results we obtained while testing this new protocol from four properties.

## 5. Experiments

### 5.1. Testbed Description

We evaluate our protocol in an indoor wireless sensor network testbed that is deployed on the twelfth floor of an office building. The testbed comprises a total of ten Arduino-based sensor nodes and multiple Linux-based gateways. Each sensor node is equipped with appropriate sensor hardware that enables it to collect data of various physical environmental modalities, such as temperature, noise and humidity. The sensor data from each node is periodically transmitted to the gateways through multiple hops, via an IEEE 802.15.4-compliant radio interface.

**Figure 1:**
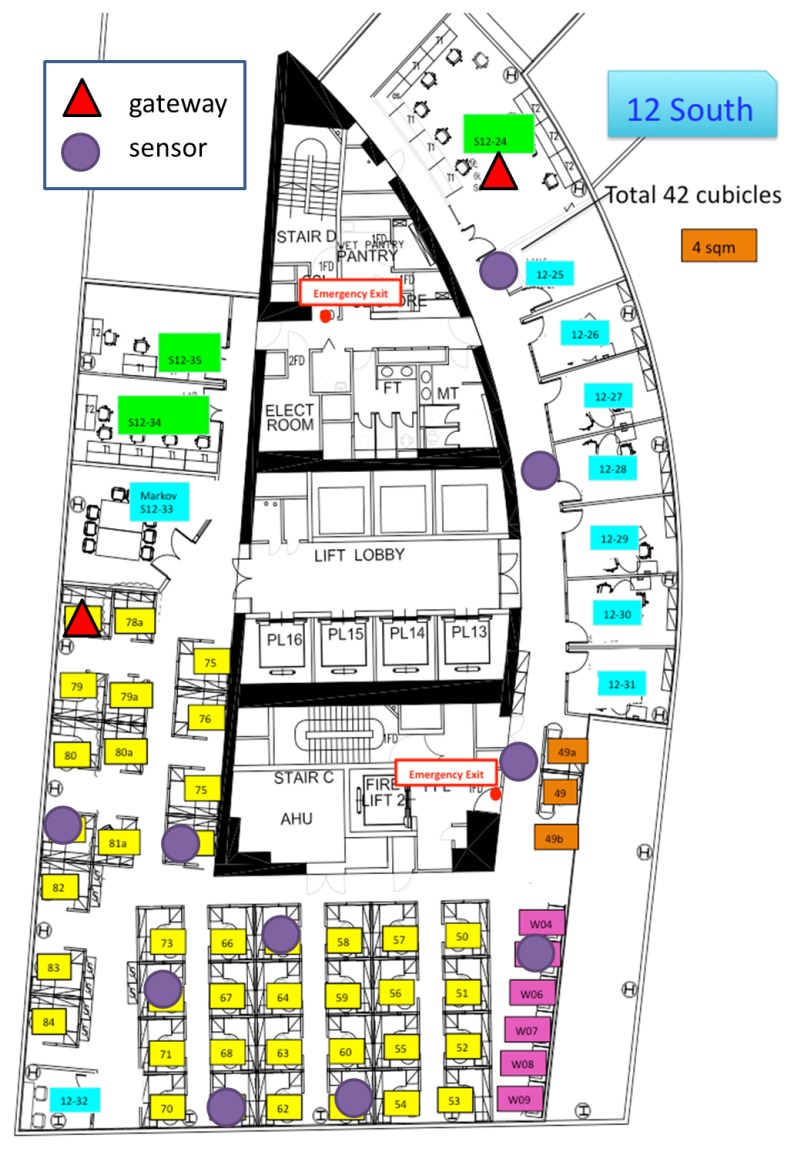
Experiment environment.

Figure 1 illustrates the location of the nodes (101 to 110 for the sensor nodes and 500 and 501 for the gateways) in the testbed, which forms a connected multi-hop topology. Due to the lack of energy harvesting sources in the building, each node is connected to a constant power supply source.

### 5.2. Traces Description

As illustrated in Figure 1, ten sensor nodes and multiple gateways are used in our experiments. Traces are collected from each of these nodes in the following way: as the nodes have limited on-board storage and solely for the testing purposes of packet trace collection, a wired USB connection between each node and a PC is provided. Whenever a node transmits or receives a message, the message content is recorded in the PC, alongside with the timestamp of the message (for both transmission and reception) and the RSSI value of the message (for reception only).

Packet traces have been collected from each of the nodes in the network, over a period of two days, and with differing test scenarios. During certain periods in time, one of the gateways may be deliberately switched off, to simulate the effect of an actual gateway that has depleted its energy in the outdoor deployment. The routing protocol is expected to recover in such scenarios by re-routing data packets to the only other existing gateway in the network.

### 5.3. Test Results

The tests are performed in a real machine with 8 GB RAM and one processor Intel i7 @3.4 GHz (Dell E7450, Round Rock, TX, USA). They are also performed through a prototype implementation of the formal approach mentioned above; this is developed in C code by using the algorithm introduced in the previous section. Firstly, we test a property commonly used for all WSN routing protocols: no routing loops.

#### 5.3.1. Property 1

A packet should not be routed to the same node more than once before reaching the intended destination. By using the syntax and semantics we mentioned before, this property can be simply formalized as:

$$\begin{array}{c} \forall_{y>x}\left(receive\left(y\right)!=message\left(x\right)\right) \end{array}$$

where receive(y) represents any message received after message *x*.

The testing results are shown in Table 1; unsurprisingly, no fail verdict has been found. The column “Total Messages” represents all of the messages we captured from each node. The columns ‘Pass’, ‘Fail’ and ‘Inconclusive’ represent the messages satisfying/not satisfying/uncertainly satisfying the requirement condition accordingly. Sometimes, there is an amount of messages concluded to be in “Total Messages”, but not relevant to the property being tested. Correspondingly, these messages are not counted in the final verdicts. That is also the reason that the total number of ‘Pass’, ‘Fail’ and ‘Inconclusive’ does not equal the ‘Total Messages’ for some properties. For this property, all of the messages in the traces satisfied the condition and passed this property. Since this property is just a simple one for all of the WSN routing protocol, for verifying the efficiency of our approach, we test more complex properties.

**Table 1:** Test results for Property 1.

Traces	Total Messages	Pass	Fail	Inconclusive
101	67,409	67,409	0	0
102	95,206	95,206	0	0
103	81,709	81,709	0	0
104	84,590	84,590	0	0
105	69,274	69,274	0	0
106	70,113	70,113	0	0
107	79,807	79,807	0	0
108	78,483	78,483	0	0
109	87,196	87,196	0	0
110	62,235	62,235	0	0
Gateway 500	60,805	60,805	0	0
Gateway 501	52,444	52,444	0	0

#### 5.3.2. Property 2

Every DATA packet will be retransmitted until one of the conditions has been satisfied: (i) up to a maximum of five times; or (ii) an ACK packet is received. This property can be differentiated into two situations, and by using the syntax and semantics we mentioned before, they are formalized as:

(i) Up to a maximum of five times:

$$\begin{array}{c} \neg \exists_{x}(repeat\left(x,\mathrm{DATA}\right)\to \exists_{t<x}\left(repeat\left(t,\mathrm{DATA}\right)\right)\to \exists_{u<x}\left(repeat\left(u,\mathrm{DATA}\right)\right)\to \\ \exists_{v<x}\left(repeat\left(v,\mathrm{DATA}\right)\right)\to \exists_{w<x}\left(repeat\left(w,\mathrm{DATA}\right)\right)) \end{array}$$

where repeat(x,DATA) is defined by:

$$\begin{array}{c} (request\left(x\right)\wedge x.type=\mathrm{DATA}\to \exists_{y<x}(request\left(y\right)\wedge y.type\\ =\mathrm{DATA}\wedge x.dataSequenceNum=y.dataSequenceNum)) \end{array}$$

(ii) An ACK packet is received:

$$\begin{array}{c} \forall_{x}(repeat\left(x,\mathrm{DATA}\right)\to \\ \exists_{z>x}\left(responds\left(z,x\right)\wedge z.type=\mathrm{ACK}\right)) \end{array}$$

Compared to Property 1, this property written in the new protocol requirements is far more complex. We test this property through the traces separately collected during two days. The results are shown in Table 2 and Table 3. These tables include the results for traces collected from different nodes (101 to 110) and gateways (500, 501).

**Table 2:** Test results for Property 2 (Day 1).

Traces	Total Messages	Pass	Fail	Inconclusive
101	67,409	22,446	0	0
102	95,206	29,552	0	0
103	81,709	32,243	0	1
104	84,590	29,128	0	0
105	69,274	23,981	0	0
106	70,113	23,983	0	0
107	79,807	26,717	0	0
108	78,483	29,330	0	1
109	87,196	26,117	0	0
110	62,235	19,837	0	0
Gateway 500	60,805	17,639	0	1
Gateway 501	52,444	14,659	0	0

**Table 3:** Test results for Property 2 (Day 2).

Traces	Total Messages	Pass	Fail	Inconclusive
101	13,203	4228	0	0
102	18,342	5496	0	1
103	15,744	5939	0	0
104	18,852	6685	0	0
105	14,711	5809	0	0
106	12,851	5811	0	1
107	18,663	6613	0	0
108	18,440	7310	0	0
109	17,985	5377	0	0
110	12,879	4125	0	1
Gateway 500	11,770	3310	0	0
Gateway 501	11,509	3230	0	0

**Figure 2:**
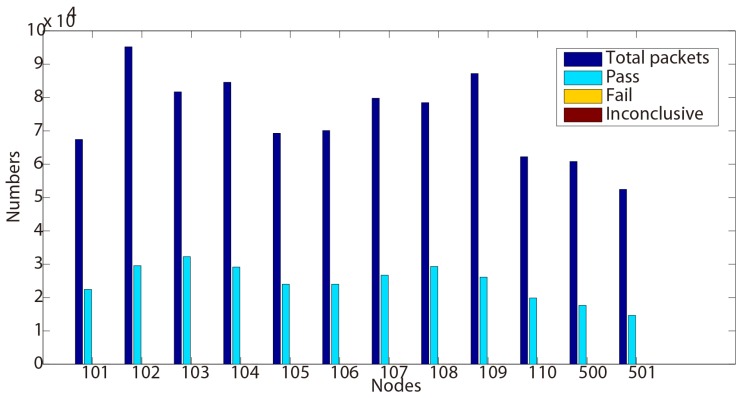
Test results for Property 2 (Day 1).

**Figure 3:**
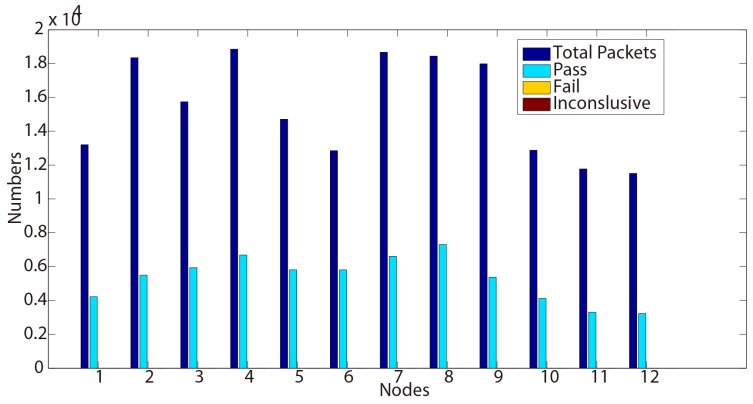
Test results for Property 2 (Day 2).

For better illustrating the results, the proportion of verdicts are also shown in Figure 2 and Figure 3. As expected, most of the traces return ‘pass’ verdicts and no ‘fail’ is detected. However, several ‘inconclusive’ verdicts can be observed from 500, 103 and 108 in Table 2 and 102, 106 and 110 in Table 3.

After the analysis of these verdicts through the collected traces, we find out that they are caused by the uncertain existence of ‘ACK’ packets. Indeed, according to the formalized formula, we search an ‘ACK’ before the received ‘DATA’ packet. Nevertheless, when the ‘DATA’ packet is received at the beginning of a trace, we cannot conclude whether there is an ‘ACK’ or not. As a result, our algorithm produces ‘inconclusive’ verdicts.

Although few ‘inconclusive’ have been observed, we may notice that no ‘fail’ verdict is raised during the testing process. This shows that this functional tested property conforms to the requirements through these nodes during two days.

Another discussion can also be opened about these ‘inconclusive’ verdicts. When testing a protocol, these kinds of verdicts could be compared to interference. The human testers have then to analyze in depth if they are not false positives/negatives or errors raised during the testing process. In our case here, these verdicts are detected on different nodes Day 1 and Day 2. The reason is that our sniffer is manually triggered, forgetting the beginning of the executions.

Therefore, in order to reduce these types of verdicts (in some experiments, depending on the protocol and the tested properties, their number can be very important), works could be performed to rewrite the formula to avoid looking in the past of the trace. Two consequences could however occur: (i) our issue is moved to the end of the trace; and (ii) the analysis complexity can be increased (or eventually decreased, as well). These aspects also make part of our future works.

#### 5.3.3. Property 3

An ACK packet from a node $v_{i}$ will be sent to another node $v_{j}$ only if both conditions are satisfied: (i) $v_{i}$ receives a DATA packet from $v_{j}$; and (ii) $v_{i}$ (currently) has a smaller metric than $v_{j}$. This property can be formalized as:

$$\begin{array}{c} \forall_{x}(response\left(x\right)\wedge x.type=\mathrm{ACK}\to \\ \exists_{y<x}\left(request\left(y,x\right)\wedge y.type=\mathrm{DATA}\wedge i.metric<j.metric\right)) \end{array}$$

We still test this property through the traces collected and used for previous properties. The results are shown in Table 4 and Table 5.

**Table 4:** Test results for Property 3 (Day 1).

Traces	Total Messages	Pass	Fail	Inconclusive
101	67,409	11,105	11,341	0
102	95,206	29,552	0	0
103	81,709	16,171	16,072	0
104	84,590	29,128	0	1
105	69,274	23,981	0	0
106	70,113	23,983	0	2
107	79,807	13,252	13,365	0
108	78,483	29,330	0	1
109	87,196	26,117	0	0
110	62,235	10,016	9821	1
Gateway 500	60,805	17,639	0	0
Gateway 501	52,444	14,659	0	0

**Table 5:** Test results for Property 3 (Day 2).

Traces	Total Messages	Pass	Fail	Inconclusive
101	13,203	2105	0	0
102	18,342	4277	0	0
103	15,744	1322	0	1
104	18,852	3651	0	0
105	14,711	431	0	0
106	12,851	80	0	0
107	18,663	3610	0	0
108	18,440	2766	0	0
109	17,985	4210	0	0
110	12,879	2045	0	1
Gateway 500	11,770	3310	0	0
Gateway 501	11,509	3230	0	0

**Figure 4:**
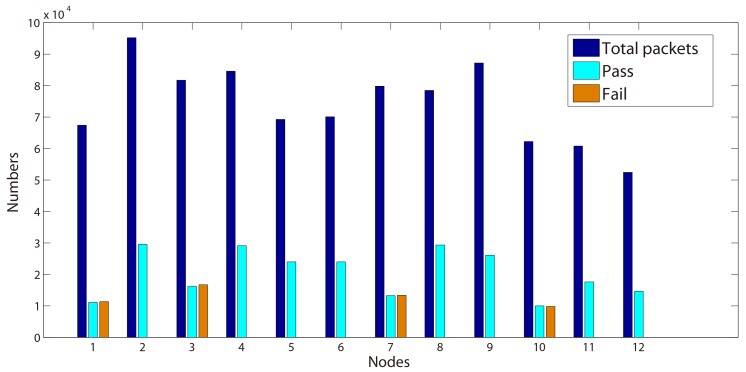
Test results for Property 3 (Day 1).

Different from no ‘fail’ verdicts in Property 2, we can observe a large number of fail verdicts in Nodes 101, 103, 107 and 110 from Table 4 and Figure 4. When we analyze these fail verdicts, they are all caused by the violation of ‘$v_{i}$ has a smaller metric than $v_{j}$’. These ‘ACK’ packets are transmitted even when $v_{i}$ has a greater metric than $v_{j}$.

This draws our attention to the implementation of these nodes. We went through the codes for Nodes 101, 103, 107 and 110 and found that there exists a configuration error in these nodes, which leads to this phenomenon.

**Figure 5:**
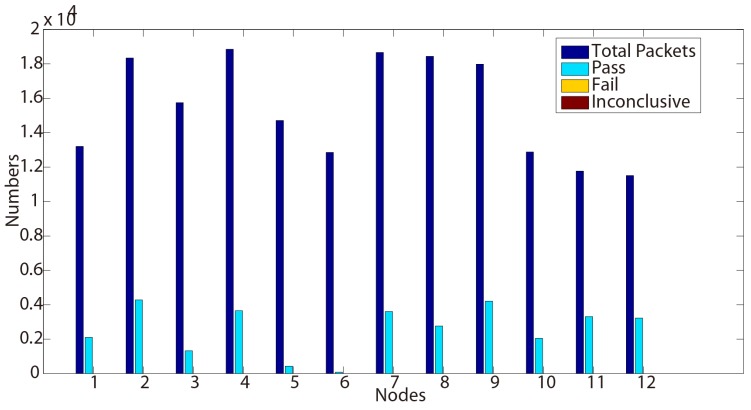
Test results for Property 3 (Day 2).

After we fixed this implementation error, there is no such fail verdict reported again on the second day testing results, as shown in Table 5 and Figure 5. Meanwhile, the ‘inconclusive’ verdicts are caused by the same reason as we mentioned in Property 2. These testing results sufficiently prove that our approach can detect errors and can help developers to find and fix existing bugs in the implementation.

#### 5.3.4. Property 4

A node $v_{i}$ with a lower metric than $v_{j}$ will not forward DATA packets to $v_{j}$. We model this property as follows:

$$\begin{array}{c} \forall_{x}\left(request\left(x\right)\wedge x.type=\mathrm{DATA}\wedge i.metric>j.metric\right) \end{array}$$

**Figure 6:**
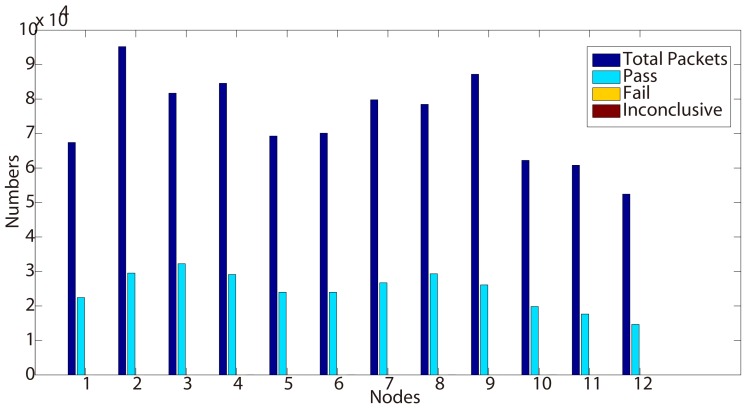
Test results for Property 4 (Day 1).

**Figure 7:**
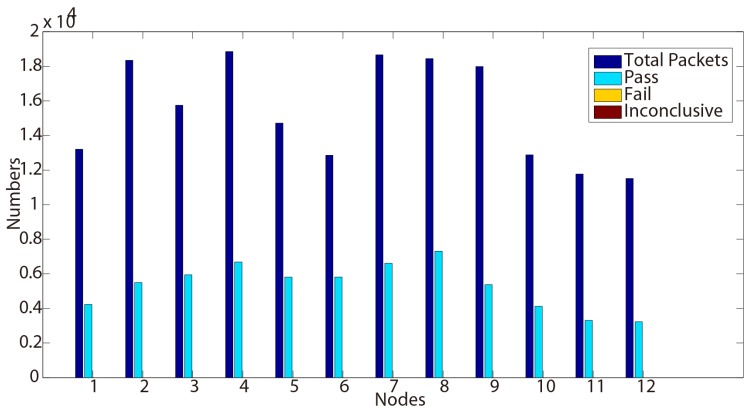
Test results for Property 4 (Day 2).

Based on the above-mentioned collected traces, we continue to test Property 4, and the results are illustrated in Table 6 and Table 7, Figure 6 and Figure 7.

**Table 6:** Test results for Property 4 (Day 1).

Traces	Total Messages	Pass	Fail	Inconclusive
101	67,409	22,446	0	0
102	95,206	29,552	0	0
103	81,709	32,243	0	0
104	84,590	29,128	0	0
105	69,274	23,981	0	0
106	70,113	23,983	0	0
107	79,807	26,717	0	0
108	78,483	29,330	0	0
109	87,196	26,117	0	0
110	62,235	19,837	0	0
Gateway 500	60,805	17,639	0	0
Gateway 501	52,444	14,659	0	0

**Table 7:** Test results for Property 4 (Day 2).

Traces	Total Messages	Pass	Fail	Inconclusive
101	13,203	4228	1	0
102	18,342	5496	0	0
103	15,744	5939	0	0
104	18,852	6685	0	0
105	14,711	5809	1	0
106	12,851	5811	0	0
107	18,663	6613	0	0
108	18,440	7310	1	1
109	17,985	5377	0	0
110	12,879	4125	0	1
Gateway 500	11,770	3310	0	0
Gateway 501	11,509	3230	0	0

During all of our testing process for this property on Day 1, no ‘fail’ verdicts have been obtained. Therefore, in order to better demonstrate that our approach and tool may detect different failures, we manually introduced some errors into several traces that will be tested on Day 2.

Three types of errors were inserted into Traces 101, 105 and 108: wrong control parts; false payload (data part or erroneous timestamps); and we moved or deleted some of the packets. As shown in Table 7, all of the errors were successfully detected. We may however note that some of the removed packets generated some ‘inconclusive’ verdicts, which is not a bad or faulty result, since this could be a real effect of packet losses.

## 6. Conclusions

This paper presents a formal approach for testing the functional requirements of a novel routing protocol used in wireless sensor networks. The routing protocol is designed by considering the inherent constraints of our smart system requirements and to cope with the drawbacks and lack of current available commercial protocols for our outdoor urban large-scale network. The main objective was therefore to test this new developed WSN routing protocol before its deployment, in a formal way, through a real testbed and without being intrusive. For that purpose, we defined a logic-based syntax and semantics to model the functional properties of the protocol. We also designed an efficient algorithm to evaluate these properties on real extracted execution traces.

Our approach has been successfully evaluated by experiments on the WSN testbed. We have shown that our formal approach has several advantages, among others: (i) the syntax and semantics are simple enough to allow any engineers (not only testers) to design their own functional properties to be tested; (ii) we may test several nodes at the same moment in a very short time; and (iii) we do not need either standardized properties or complete formal models to test a protocol; this is highly convenient while testing a new protocol during its development and deployment periods.

For future work, we will study the rewriting of some specific functional properties and the impacts on the testing process. We will apply and assess our approach on our large-scale outdoor deployed WSN focusing on the reliability of our algorithm, our protocol and specific behaviors between gateways.
